# Does the volume overload exaggerate the severity of mitral regurgitation in patients with decompensated heart failure?

**DOI:** 10.3906/sag-2001-220

**Published:** 2020-10-22

**Authors:** Göktuğ SAVAŞ, Ömer ŞAHİN, Mustafa YAŞAN, Uğur KARABIYIK, Nihat KALAY, Ali DOĞAN, Ferhan ELMALI, Abdurrahman OĞUZHAN

**Affiliations:** 1 Department of Cardiology, Siyami Ersek Thoracic and Cardiovascular Surgery Center Training and Research Hospital, İstanbul Turkey; 2 Department of Cardiology, Kayseri Training and Research Hospital, Kayseri Turkey; 3 Department of Cardiology, Erciyes University School of Medicine, Kayseri Turkey; 4 Department of Biostatistics and Bioinformatics, Erciyes University School of Medicine, Kayseri Turkey

**Keywords:** Functional mitral regurgitation, BNP, volume overload, heart failure, echocardiography

## Abstract

**Background/aim:**

Diagnosing and managing functional mitral regurgitation (MR) is often challenging and requires an integrated approach including a comprehensive echocardiographic examination. However, the effects of volume overload on the echocardiographic assessment of MR severity are uncertain. The purpose of this study was to weigh the effects of volume overload in the echocardiographic assessment of MR severity among patients with heart failure (HF).

**Materials and methods:**

Twenty-nine patients with decompensated HF, who had moderate or severe MR, were included in the present study. The volume status and the N-terminal pro-B-type natriuretic peptide (proBNP) levels were recorded and the echocardiographic parameters were assessed. After the conventional treatment for HF, the proBNP levels and the echocardiographic parameters were assessed again.

**Results:**

The mean age of the patients was 72 ± 9 years and the average hospitalization time was 10.9 ± 5.9 days. Between the beginning and the end of the treatment, there were significant reductions in the effective regurgitant orifice area (EROA) (0.36 ± 0.09 cm^2^ to 0.29 ± 0.09 cm^2^, P < 0.001), vena contracta (VC) (P < 0.001), the regurgitant volume (RV) (P < 0.001), and systolic pulmonary artery pressure (sPAP) (P < 0.001).

**Conclusion:**

This is the first study to investigate the relationship of changes in severity of MR with volume-load by monitoring the proBNP levels among patients with HF. The present results demonstrated that volume reduction, as evidenced by a decline in the proBNP levels, was accompanied by a marked reduction in the EROA, VC, and the RV among patients with left ventricular dysfunction.

## 1. Introduction

Functional mitral regurgitation (MR) occurs in approximately one-third to one-half of patients with cardiomyopathy and contributes to the progression of ventricular dysfunction [1,2]. 

The presence of functional MR also serves as a marker of worse outcomes in patients with heart failure (HF). Without intervention, survival rates at five years were about 40% in those with moderate or severe MR [2]. Current guidelines provide detailed recommendations for the treatment of patients with MR in HF, including cardiac resynchronization therapy and mitral valve surgery [3,4]. Recently interventional approaches to MR in patients with left ventricular dysfunction have shown to have a potential role [5]. Use of these invasive procedures in such cases confers a significant operational risk and cost; therefore, precise evaluation of secondary MR severity is quite important.

The severity assessment of MR is often made based on echocardiographic criteria. The integration of multiple parameters, including vena contracta width (VC), the effective regurgitant orifice area (EROA), the regurgitant volume (RV), and adjunctive signs are important for accurate assessment of MR severity [6]. Although functional MR is dynamic in nature [7,8], the effects of volume overload on MR severity quantitation have been less clearly identified. 

B-type natriuretic peptide (BNP) is a cardiac neurohormone synthesized in the cardiac ventricles as a result of increased wall stress. Diastolic stretch induces cardiomyocyte proBNP expression in volume-loaded conditions such as MR [9–11]. Furthermore, serum proBNP has been suggested to be an indicator of preload [12]. In the present study, therefore, we monitored the serum proBNP levels as a marker of preload and we aimed to describe the effects of volume overload in the echocardiographic assessment of MR severity among patients with HF.

## 2. Materials and methods

### 2.1. Study population

Twenty-nine patients with decompensated HF who had moderate or severe MR were included in the present prospective study between January 2016 and June 2016. The study protocol was approved by our local institutional ethics committee and all participants provided informed written consents. The exclusion criteria of the study were as follows: poor image quality, recent myocardial infarction (within three months), structural mitral valve abnormalities including mitral valve repair or replacement history, moderate or severe aortic valve disease, coronary revascularization schedule, hypertrophic cardiomyopathy, primary pulmonary hypertension, and intracardiac shunts.

All included subjects who were hospitalized for decompensated HF were consecutively enrolled. Baseline clinical information including echocardiographic parameters, volume status, and proBNP levels were recorded. All participants received a continuous intravenous infusion of furosemide during their hospital stay. After the conventional treatment for HF, proBNP levels and the echocardiographic parameters were again assessed on the day of discharge. Follow-up vital status information was conducted.

### 2.2. Echocardiographic analysis

Blood pressures were measured using an appropriate cuff in all cases before the echocardiographic examination, and if their systolic blood pressure (SBP) was ≥140 mmHg or diastolic blood pressure (DBP) ≥90 mmHg, captopril was orally administered in order to control the blood pressure. After the regulation of blood pressure, the echocardiographic evaluation was assessed.

Echocardiography was performed using a commercially available instrument (Vivid 7® GE Medical System, Horten, Norway) with a 2.5-MHz transducer by an experienced echocardiography specialist, who was blinded to all patients. All images were stored for subsequent analysis and then a single, blinded observer performed offline analysis. To determine intraobserver variability, stored images of 15 randomly selected patients were reanalyzed one month later by the same observer.

All patients underwent a comprehensive M-mode, two-dimensional (2D), and color Doppler study. When the patients were in sinus rhythm, three cardiac cycles were averaged for all measurements. For patients in atrial fibrillation, five cardiac cycles were averaged. Left ventricular dimensions were assessed from parasternal long-axis views using two-dimensional-guided M-mode with the leading-edge method at end-diastole and -systole. The left ventricular ejection fraction was calculated by the modified Simpson’s rule as recommended by the European Association of Cardiovascular Imaging [13]. The MR was graded quantitatively in an integrative fashion calculating VC, EROA, and RV as described in the guidelines [4,14]. The VC width was imaged in multiple views perpendicular to the commissural line to obtain the largest regurgitant jet size, and the width of the narrowest portion of the jet was then measured [15]. The EROA was obtained using the proximal isovelocity surface area (PISA) method through color flow mapping in patients with central MR jets. However, in the case of eccentric or multiple MR jets, EROA calculation was made using the pulsed wave Doppler method. The RV was estimated as EROA multiplied by the velocity time integral of the regurgitant jet [16]. The systolic pressure gradient peak from the right ventricle to the right atrium was assessed using a simplified Bernoulli equation and then the right atrium pressure was added to obtain the sPAP [13].

### 2.3. Statistical analysis

The SPSS 22.0 (IBM Corporation, Armonk, NY, USA) software package was used for statistical analysis. Categorical variables are presented as number and percentage. Continuous variables with normal distribution are presented as mean ± SD; non-normal variables are reported as median. The Shapiro-Wilk test was used to determine the normality of distribution of datasets. The mean comparison of parameters before and after heart failure treatment were done with Wilcoxon signed-rank test. And the mean comparison of parameters between patients received inotropic treatment or not were done with Mann-Whitney U test. The relationship between two quantitative variables was analyzed using Spearman’s test. The frequencies of categorical variables were compared using Pearson χ2 test, when appropriate. A P-value <0.05 was considered statistically significant. Intraobserver variability was evaluated by means of the intra-class correlation coefficient.

## 3. Results

The baseline characteristics of the patients are shown in Table 1. The mean age of the patients was 72 ± 9 (range, 52–87) years, and 48.2% were male. The average length of the hospital stay was 10.9 ± 5.9 days. At the beginning of hospitalization, continuous IV inotrope was used as a bridge to standard medical therapy in 14 patients (48.2%). Dobutamine was ordered at 5–15 mcg/kg/min for nine of these patients, and dopamine was ordered at a moderate dose (5–10 mcg/kg/min) for four patients. One patient received high-dose dopamine (>10 mcg/kg/min) at the day of admission. The doses were titrated according to patient responses (Table 2 shows the difference of medication between on admission and at discharge)

**Table 1:** Baseline characteristics of the patients.

Parameters	Patients (n = 29)
Age (years)	72 ± 9
Males	14 (48.2%)
Body mass index (kg/m2)	30.8 ± 5.2
Hypertension	20 (68.9%)
Coronary artery disease	16 (55.1%)
Diabetes mellitus	9 (31%)
Atrial fibrillation	10 (34.4%)
Hyperlipidemia	11 (37.9%)
Smoking	8 (27.5%)
Systolic blood pressure, mmHg	112.9 ± 12.3
Diastolic blood pressure, mmHg	67.3 ± 5.4
Heart rate, per minute	94.3 ± 16.8
Type of mitral regurgitation jet	
Central	23 (79.3%)
Eccentric	6 (20.6%)
Laboratory results at admission	
Hemoglobin (g/dL)	12.9 ± 2.09
Creatinine (mg/dL)	1.19 ± 0.3
Low- density lipoprotein (mg/dL)	85.6 ± 23.3
proBNP, pg/mL (median)	14338

Data: mean ± SD. proBNP = pro-B-type natriuretic peptide.

**Table 2:** The difference in medication between admission and discharge.

Medication	On admission, n (%)	At discharge, n (%)
Angiotensin-converting enzyme inhibitor/angiotensin receptor blocker	14 (48.2%)	20 (68.9%)
β-blocker	16 (55.1%)	22 (75.8%)
Digoxin	4 (13.7%)	7 (24.1%)
Aspirin	16 (55.1%)	14 (48.2%)
Warfarin	5 (17.2%)	10 (34.4%)
Aldosterone antagonist	10 (34.4%)	16 (55.1%)
Furosemide	20 (68.9%)	21 (72.4%)
IV inotrope therapy Dobutamine Dopamine	14 (48.2%)9 (31%)5 (17.2%)	---

Six patients died during follow-up. Pump failure was the cause of death in three patients. Acute renal failure resulted in death in two patients and one patient died of septic shock due to catheter infection. Median New York Heart Association (NYHA) class was IV at baseline, and it improved to class III after treatment. There was an average weight loss of 5.8 ± 1.6 kg/participant. Thyroid function, hepatic enzymes, and the blood glucose levels of the patients were in the normal range, and the mean hemoglobin level was 12.9 ± 2.09 g/dL. The mean SBP was 112.9 ± 12.3 mmHg at admission and 110 ± 13.8 mmHg at the discharge (P = 0.023). The mean DBP was 67.3 ± 5.4 mmHg at admission and 66 ± 6.1 mmHg at the conclusion of the therapy (P = 0.225). The average iv furosemide dose was 63.4 ± 19.04 mg/day, and the median (interquartile range) proBNP level was 14,338 pg/mL (6068–35,000 pg/mL), which decreased after medical therapy (8819 pg/mL).

**Figure 1:**
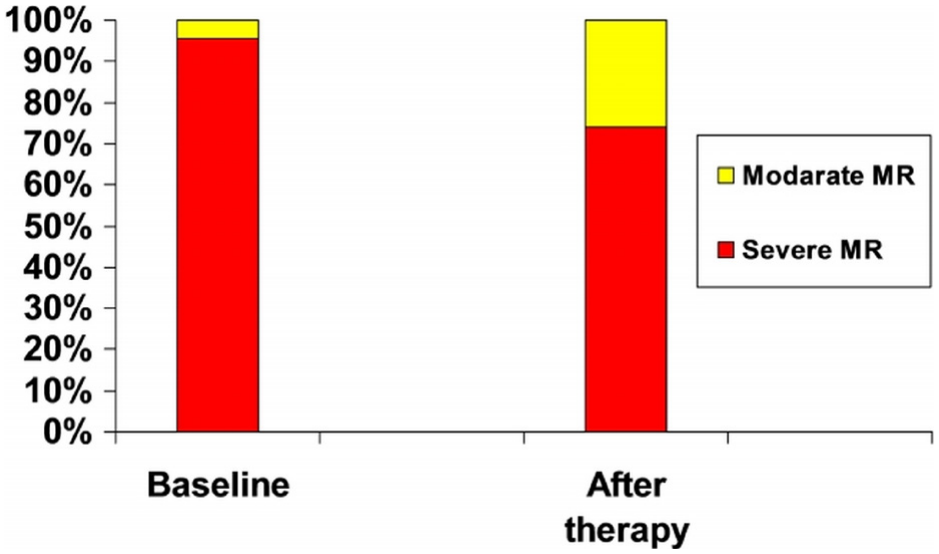
MR severity evaluated by transthoracic echocardiography at baseline and after therapy. MR severity compared from baseline to the last visit with an average length of hospital stay of 10.9 ± 5.9 days. Results are matched and presented for patients who did not die; data are given in percentage.

**Figure 2:**
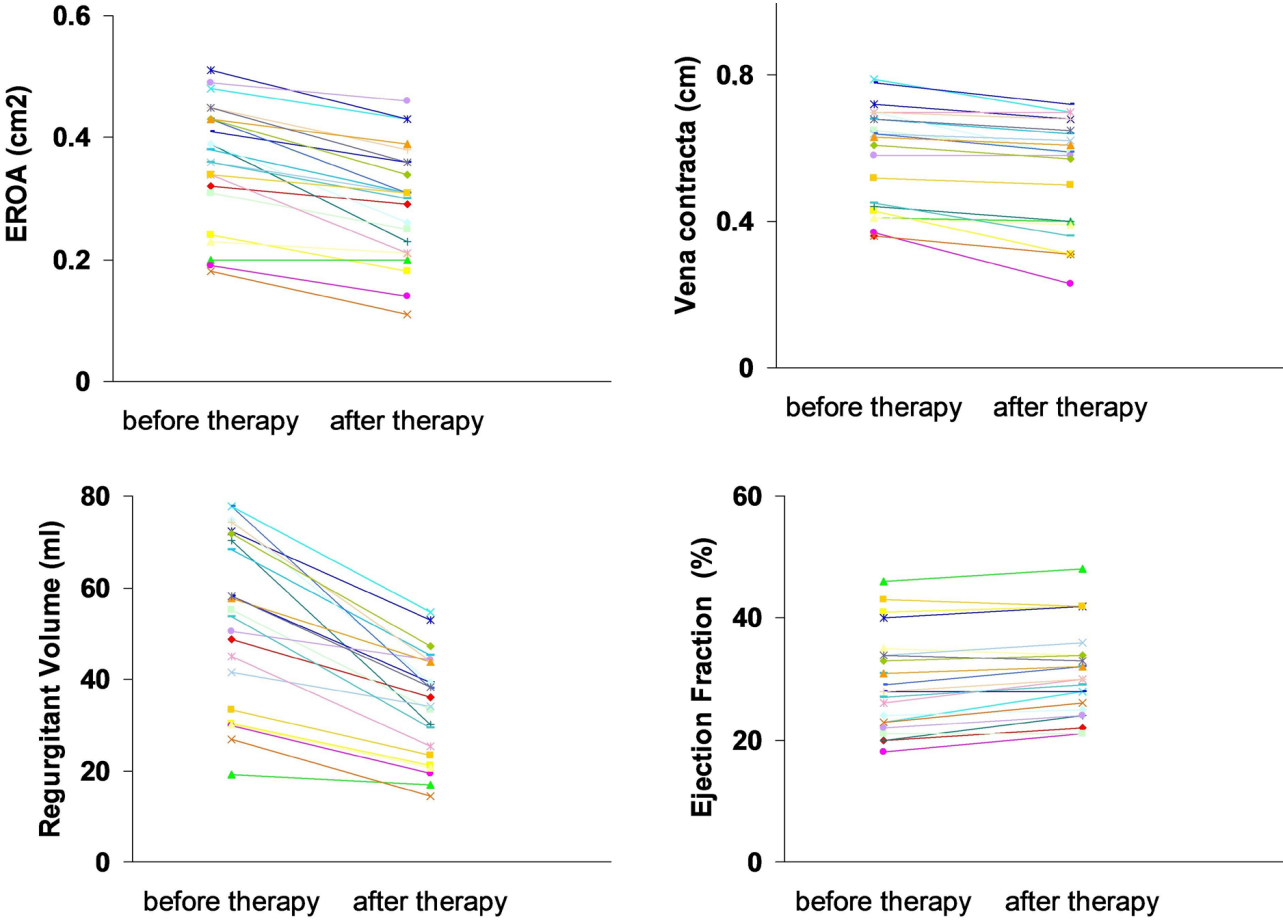
Individual values for effective regurgitant orifice area (EROA), vena contracta, regurgitant volume and ejection fraction at baseline and after the therapy. Results are matched and presented for patients who did not die.

One-quarter of the patients who had severe MR at baseline were diagnosed with moderate MR after therapy (Figure 1). Between the initiation and the conclusion of the therapy, there were significant reductions in EROA (0.36 ± 0.09 cm2 to 0.29 ± 0.09 cm2, P < 0.001), VC (0.57 ± 0.14 cm to 0.52 ± 0.15 cm, P < 0.001), RV (53.2 ± 18.4 mL to 34.3 ± 11.5 mL, P < 0.001), left ventricular dimensions, and sPAP (61.5 ± 12.2 mmHg to 51.1 ± 7.9 mmHg, P < 0.001) (Table 3, Figure 2). The ejection fraction increased from 29.4 ± 7.8 % prior to therapy to 31.08 ± 7.2 % after therapy (P < 0.001). There were not significant differences in the changes of echocardiographic parameters between patients who did and did not receive IV inotrope therapy.

In addition, we have found a significant positive correlation between the decrease in proBNP level and the reduction in EROA (r = 0.447, P = 0.032). However, there was no correlation between the decrease in proBNP level and the reduction in RV, and vena contracta (r = 0.167, P = 0.447 and r = 0.027, P = 0.904, respectively). In an investigation of the relationship between the weight loss of the patients and alteration of EROA, RV and vena contracta, there was no correlation between weight loss and these parameters (r = 0.124, P = 0.573; r = 0.210, P = 0.336 and r = 0.401, P = 0.058, respectively) (Table 4).

**Table 3:** The difference in echocardiographic and laboratory measurements between the initiation and conclusion of the therapy.

Measures	Pretreatment	Posttreatment	P-values
Echocardiography			
LVEDD, cm	6.42 ± 0.7	6.2 ± 0.6	<0.001
LVESD, cm	5.1 ± 0.7	4.9 ± 0.6	<0.001
EF %	29.4 ± 7.8	31.08 ± 7.2	<0.001
sPAP, mmHg	61.5 ± 12.2	51.1 ± 7.9	<0.001
EROA, cm2	0.36 ± 0.09	0.29 ± 0.09	<0.001
Vena contracta, cm	0.57 ± 0.14	0.52 ± 0.15	<0.001
RV, mL	53.2 ± 18.4	34.3 ± 11.5	<0.001
Laboratory			
proBNP, pg/mL ,median [25%–75%]	14338 [12690-19202]	8819 [4120-12405]	<0.001
Creatinine, mg/dL	1.19 ± 0.3	1.33 ± 0.2	0.016

Data are mean ± SD. proBNP values are presented as the median and 25%–75% quartile. LVEDD = Left ventricle end-diastolic diameter; LVESD = Left ventricle end systolic diameter; EF = Ejection fraction; sPAP = Systolic pulmonary artery pressure; EROA = Effective regurgitant orifice area; RV = Regurgitant volume; proBNP = pro-B-type natriuretic peptide.

**Table 4:** The correlation analyses between the changes in volume status and echocardiographic measures of mitral regurgitation severity.

Measurements	EROA	RV	Vena contracta
proBNP, pg/mL	r = 0.447P = 0.032	r = 0.167 P = 0.447	r = 0.027P = 0.904
Weight loss in kg	r = 0.124P = 0.573	r = 0.210P = 0.336	r = 0.401P = 0.058

EROA = Effective regurgitant orifice area; RV = Regurgitant volume; proBNP = pro-B-type natriuretic peptide.

## 4. Discussion

This study of the patients with advanced HF, who had moderate or severe MR, demonstrated that volume reduction with diuretics, as evidenced by a decline in proBNP levels, was accompanied by a marked reduction in EROA, vena contracta, and RV. This reduction in MR severity appeared to result from a decrease in left ventricular end-diastolic dimensions consistent with a decrease in volume load on the left atrium and left ventricle. Furthermore, the elimination of volume overload with therapy resulted in a substantial decrease in sPAP, but no significant change in blood pressure.

In HF, progressive LV dilation and remodeling culminate in impairment of leaflet coaptation and decreased valvular closing forces, resulting in secondary MR [5]. These pathologic changes are dependent on loading conditions. Secondary MR is, therefore, dynamic in nature [7]. The hemodynamic effects of exercise and various inotropic and vasodilator agents in patients with secondary MR have been studied [17, 18]. In an earlier study, Keren et al. looked at patients with severe congestive HF and showed that isometric exercise resulted in an increase in MR volume. They also revealed that both nitroglycerin and dobutamine therapy led to a reduction in MR in which mitral insufficiency was greatest [17]. Subsequent studies have described distinctive responses to nitroprusside and dobutamine therapy. Nitroprusside resulted in a reduction in MR, and dobutamine led to improved cardiac output with a variable effect on the degree of MR in color Doppler echocardiography [2,19]. These conflicting results could be attributed to the inclusion of different causes of MR. Studies including patients who had a diseased mitral valve apparatus such as rheumatic heart disease or mitral annular calcification, might fail to show the benefit of vasodilator therapy because these patients are thought to have a fixed orifice, whereas patients with MR caused by dilated cardiomyopathy, by contrast, are thought to have a dynamic orifice [5].

Although prior studies have described a recovery in the color jet area of MR and EROA in patients treated with vasoactive drugs, which produced a reduction in afterload [17, 18], the present study is the first to examine the relationship of changes in volume load and MR severity, while monitoring proBNP levels. More precisely, our study evaluates the magnitude of this change by using quantitative parameters of MR. The decrease in VC, RV, and EROA was not associated with any significant changes in the SBP. A significant relationship was found between the decrease in proBNP levels and the reduction in EROA. However, an optimal cut-off point of proBNP for predicting alterations of EROA could not be identified because of the limited number of patients recruited for the study whose proBNP levels were heterogeneous. Use of proBNP levels to predict a decrease in alteration of EROA, VC, and RV in patients with HF remains unproven and further studies with large sample sizes are needed to clarify this issue.

Reduction of MR is likely to be predominantly related to improvement in the leaflet coaptation at lower left ventricular volumes. In addition, a reduction in left ventricular volumes may improve the relationship between the subvalvular apparatus and valve leaflets while also decreasing ischemia in patients who have coronary artery disease. Indeed, it is difficult to determine the relative contributions of changes in the annulus and the subvalvular apparatus to this effect because the mechanisms underlying MR are complex [5].

One of the remarkable findings of the present study is that LVEF increased significantly after heart failure therapy. This recovery would necessarily suggest that LV unloading might lessen sphericity; intrinsic contractility and especially cardiac mechanics have been restored; protein expression has been normalized; and the neurohormonal activation has been interrupted [20]. Previous studies also revealed an improvement in left ventricular ejection fraction among patients with HF after medical therapy [21–23]. Moreover, Maurer et al. reported both a decrease in heart rate and an improvement in chamber contractility associated with beta blocker therapy within 2 weeks in patients with HF, and these parameters were found to be the primary factors contributing to the increase in ejection fraction [23].

The incidence of HF increases with age, and thus the number of patients with HF who have MR, requiring hospitalization or intervention will rise sharply in the next decades [24,25]. Recently, interventional approaches to MR in patients with left ventricular dysfunction have been shown to have a potential role. MitraClip therapy (involves a significant average cost) for secondary MR can improve symptoms, functional capacity, quality of life, and may induce reverse LV remodeling in high-risk patients, who otherwise would have limited therapeutic options [26–27]. Thus, the correct evaluation of secondary MR severity is quite important. Overestimating the MR carries all the risks of aggressive treatment and involves high financial cost. In our study population, one-quarter of the patients who had severe MR at baseline were diagnosed as having moderate MR after therapy. Therefore, we recommend that volume status should be considered when making the diagnosis of severe MR. 

Limitations: The major limitation of this study is the small sample size. The calculation of mitral regurgitant flow makes several assumptions regarding a round regurgitant orifice and in the technique of the proximal isovelocity surface area as previously described [16]. Heart failure therapy was not randomized and it would not be possible to attribute the effects to a specific drug. Our findings may not apply to patients with other etiologies of MR, such as rheumatic valvular disease.

In conclusion, the present results demonstrated that volume reduction, as evidenced by a decline in proBNP levels, was accompanied by a marked reduction in EROA, vena contracta, and RV in patients with left ventricular dysfunction. Therefore, volume status should also be considered when making decisions about interventional approaches in these patients. Further studies are needed with larger sample sizes to verify this issue.

**Conflict of interest**

The authors have no conflict of interest to declare.
